# Addressing the Needs of Preclinical Student Researchers in the SCI Community to Advance Communication and Education

**DOI:** 10.1523/ENEURO.0040-23.2023

**Published:** 2023-05-03

**Authors:** Shayna Singh, Jeremy Weinberger, Micaela O’Reilly, Andrey Borisyuk

**Affiliations:** Marion Murray Spinal Cord Research Center, Department of Neurobiology and Anatomy, Drexel University, Philadelphia, PA 19129

**Keywords:** data sharing, diversity, equity, graduate students, spinal cord injury, variability

## Introduction

As basic and preclinical scientists, graduate students and trainees emphatically support the importance of both hearing and understanding the perspectives of individuals living with spinal cord injury (SCI); however, it is much more complex than most realize. Establishing avenues of communication among individuals with SCI, caretakers, and/or researchers is paramount to the success of our efforts in becoming well rounded researchers that are knowledgeable about principles of human well being. Here, we discuss the divides between researchers and individuals living with SCI that often create misunderstandings. By expanding on two topics within these divides, we seek to highlight the main message of this commentary from the student perspective: there is a need within the SCI research community for better data-sharing practices and communication among preclinical researchers, clinicians, and individuals living with SCI. First, we will discuss the diversity of data collection and sharing within the research realm. To continue to produce relevant research, we must increase communication among researchers to boost accessibility with data sharing. Then, we will discuss relevant diversities in the SCI human population as we young basic research scientists understand them to exist within the clinical setting. Such diversity is evident when diving into an individual’s background relating to culture, socioeconomic status, and education, which may all impact the care one seeks after sustaining an injury. By considering better data-sharing practices as well as the variability and diversity within the human SCI condition, we and researchers at every level in their careers may better appreciate the complexity of the human condition after SCI and therefore ask questions with higher relevancy and value in our own research.

## Data sharing

For graduate students, a major focus of graduate training is publishing work in scientific journals as this is our formal path to most broadly communicating our research to the SCI community. Unfortunately, a substantial amount of generated data may never be published or shared. This can be motivated by high publishing standards, grant proposal aspirations, and the desire to protect intellectual property. Furthermore, trainees in academia are rarely encouraged to engage in data-sharing practices focused on nonscientific audiences beyond academia. This represents one example of a clear disconnect between scientific research and individuals living with SCI. As basic researchers, it can be challenging to even conceptually consider how experimental results can directly service or connect to the current population of individuals living with SCI. However, this is an important feature of SCI research that must be better incorporated into our academic research programs. It should be a core responsibility of basic and preclinical researchers to connect their work to the current population of individuals living with SCI as directly as possible. At the same time, scientific researchers have the responsibility to present high-quality, verifiable, and reproducible findings in ways that prevent false narratives and minimize false hope for individuals living with SCI and their families. Clearly, data sharing is a complex duty for researchers.

Across SCI research laboratories, there is a lack of consistency in data collection practices. While this encourages independent critical thinking, it means our research community has much work to do in the pursuit of disseminating our data to the public in a consumable way. Great strides have been made toward promoting findable, accessible, interoperable, and reusable (FAIR) data practices within the basic and preclinical SCI research community in the form of a virtual open data repository (Torres-Espín et al., 2022). The data from research laboratories that have been formatted, submitted, and ultimately uploaded into this FAIR database is then available for free download by anyone who has established a free account. This database is being advertised to clinicians, individuals living with SCI, and caretakers within the SCI community who may directly or indirectly benefit from information. We, as members of the SCI research community, must continue to take advantage of opportunities for outreach beyond the laboratory, such as at conferences and retreats, where we can make meaningful connections with clinicians, individuals living with SCI, and other researchers. Our communities can benefit greatly from a better understanding of the needs of modern clinicians and individuals living with SCI, which are increasingly diverse and individualized. For example, the accessibility and needs of a spinal cord-injured individual living with SCI in a rural community can vary drastically from those of someone in an urban setting. This diversity must be understood and considered in our pursuit of greater basic knowledge and more advanced therapies that positively impact individuals living with SCI.

## Diversity and Accessibility Within the Current Population of Individuals Living with SCI

Within the current population of individuals living with SCI, there is a wide array of diversity within personal, family, and socioeconomic status (Khazaeipour et al., 2018). Because of this, the population pool can have varying needs, goals, and desires that can have a substantial impact on care and quality of life (Scheel‐Sailer et al., 2017). Furthermore, the efforts of researchers, physicians, and caretakers can obfuscate issues of data sharing, reproducibility, and consistency, resulting seemingly in a standstill in progress. As trainees representing the next generation of researchers in the field, we want to promote a better understanding of our work toward improving peoples’ lives, especially those who may be directly impacted by our research in the future. It is increasingly important to continually expand communication beyond the SCI research community. For these reasons, we are advocating for stronger direct connections between researchers and the members of the clinical SCI community. Specifically, we want to highlight the diversity of people with SCI and differences in accessibility to the most informed care among individuals because we believe that, in strengthening connections, individuals with SCI are the ones who should dictate what the priorities are for all members of the SCI community.

The impact that an individual’s education level, marital status, and occupation can have on their treatment plan is astronomical, which is further multiplied by the impact of these variables within one’s immediate family (Khazaeipour et al., 2018). Difficulties with choosing proper care, understanding treatment options, or even vocalizing their needs can make matters worse by adding to burdens beyond the injury itself (Scheel‐Sailer et al., 2017). Such diversity among individuals living with SCI creates a difficult setting for physicians, researchers, and caretakers to appropriately support, as each individual living with SCI necessitates individualized care and attention.

Early after sustaining a SCI, one of the primary questions from individuals living with SCI is centered on “How long will it take for me to recover?”, quickly followed by “How will we afford this?” As scientists, we are peripherally aware of the mental and financial burden that accompanies SCI, but rarely is there the opportunity to discuss and comprehend the full scope of injury. Furthermore, these burdens are often exacerbated by a lack of knowledge surrounding treatment options, and the viability and even feasibility of such risky ventures. These concerns have been voiced by some individuals living with SCI as having little to no knowledge about what clinical trials are available.

Embedded within all of this, ultimately, is a lack of adequate communication. Whether it is among caretakers, physicians, researchers, or individuals living with SCI, the inadequacy of communication is similarly exhibited across these issues. Relevant and updated communication is paramount as recovery, rehabilitation, and autonomy begin to take shape. Importantly, education and access to resources can change the perspectives of individuals living with SCI and their experience when taking control of their health care plans following SCI (Scheel‐Sailer et al., 2017). This should not be one sided, however, and there must be a balance between the learning experience for individuals living with SCI as well as those researching their ailments and treating or caring for them. As a preclinical researcher, these factors may seem daunting to consider. Where does one begin? We propose that in-person events focused on learning about and discussing the human condition are extremely valuable first steps. The points mentioned in this commentary can serve as talking points among clinicians, researchers, and individuals living with SCI to lead, hopefully, to a rich discussion of priorities, goals, and a broadened outlook on SCI research.

## Conclusion

Even nonclinical research programs can sponsor events focused on the human experience after SCI. A good starting point for scientific researchers is to invite clinicians and individuals living with SCI to give or attend a presentation within your distinct research community. This can be as simple as a guest at a laboratory meeting or a speaker at a department-wide seminar. In 2022, the Marion Murray Spinal Cord Research Center at Drexel University held its first annual National Institutes of Health T32-sponsored retreat, entitled “Human Perspectives of Spinal Cord Injury.” It was attended by students, faculty, and individuals living with SCI. Jacob Chalfin, Chair of the SCI Advisory Committee for the state of Pennsylvania, gave the featured presentation entitled “A seat at the lab bench, leveraging the lived experience,” in which he discussed living with SCI in a way that trainees do not hear in classes, laboratories, or even at many conferences. As basic research scientists, we were invited to broaden our perspectives throughout Jacob’s talk. Such exposure to different members of the SCI research community can then grow into more regular meetings, further internal discussions within the laboratory, and even long-term collaborations. At the very least, this exposure will provide a new perspective on the research we perform, which can oftentimes feel infinitesimal within the broader SCI community. It is important to recognize the ground-breaking program launched by Unite2Fight Paralysis called “Lab Rats,” which is designed to bring individuals with SCIs into SCI research laboratories to help address many of the deficiencies we noted in our training programs as basic science researchers.

As scientists, we recognize that increasing accessibility to research within the current population of individuals living with SCI and data transparency in general in the SCI research community represent goals worth striving toward. Currently, these endeavors are challenging within the existing frameworks of academia. However, reaching out to clinicians and individuals living with SCI is a relatively straightforward way to begin to form a more comprehensive network. This can be initiated by young scientists in research programs as well as more senior researchers and investigators, as preclinical researchers at any level in their career may benefit from connecting to the human perspective of SCI. These connections can only strengthen the field at large, and can result in better understanding on all sides of the major goals and limitations associated with SCI research.

## Synthesis

Reviewing Editor: Christine Portfors, Washington State University Decisions are customarily a result of the Reviewing Editor and the peer reviewers coming together and discussing their recommendations until a consensus is reached. When revisions are invited, a fact-based synthesis statement explaining their decision and outlining what is needed to prepare a revision will be listed below. The following reviewer(s) agreed to reveal their identity: NONE.

Synthesis:

As written the commentary is quite ambitious. It attempts to present two arguments: 1) the need for data sharing between interested parties (researchers, clinicians, and the SC injured community) and increased accessibility; and 2) the need to adress the heterogeneity within the human SC injured population. This ambitiousness unfortunately makes for a complicated and hard to follow narrative. Indeed, sometimes it appears that written passages jump from one idea to another and there is a lack of structured thought. With that being said, the reviewers think the core message that the authors are trying to make is important and should be addressed. Perhaps, not in this specific way. The reviewers recommend the authors simplify the message. This will improve the clarity of what they are trying to advocate for. Please clarify the core message.

Concerns:

1) The authors should refrain from using the word “patient” when referring to the general SC injured population. This can be a trigger word. In some context, it may be appropriate but to generally refer to the entire SC injured population as “patients” can come across as dehumanizing and in a submissive role which is not the intention.

2) An argument can be made that it‘s not just “young” scientists that support the ideas you advocate for in the commentary. There are senior investigators who may have the same desires. With that being said, how do we synergize or build momentum across the entire research spectrum?

## Author Response

We thank the reviewers for taking the time to review our manuscript and for providing insightful, constructive feedback. We feel this feedback only enhances our commentary and we therefore found it to be very valuable. We are grateful especially for the feedback on word choice, as we would like to be as sensitive and inclusive as possible in our writing. Below we have detailed our revisions as they specifically relate to the reviews. Need for structure and a clear core message: We are grateful to the reviewers for their helpful feedback on the lack of clarity in our writing. We understand that our undertaking is ambitious in addressing two separate arguments within our core message. We also completely understand the importance of highlighting this core message in our writing, so we greatly appreciate the request to clarify this. To address these reviews, we have directly stated our core message in the introduction paragraph, in a way that mirrors the writing in our conclusion text. We think this unifies our purpose in making these arguments. In addition, to correct the abrupt jumps between topics, we altered the transitions between main sections in highlighted writing. We have streamlined our writing addressing heterogeneity within the human SCI population by removing the topic of “variability,” and focusing on diversity and accessibility. We did this to tighten our writing and more closely connect this section to our core message. The word “patient,” when referring to the general SCI injured population: We thank the reviewers for highlighting this concern, and for understanding that it is not our intention to dehumanize individuals or deem them submissive. We have replaced all uses of the word “patient” with “individuals living with SCI.” This phrasing better captures our intent, which is to highlight the breadth of variability and diversity in humans living with SCI, whether they are in the clinic or not. The word “young,” and the need to include senior scientists and clinicians with similar desires: We thank the reviewers for highlighting this concern. We certainly agree that we should be more intentional in including researchers who are not beginning in their careers. Except for when referring to ourselves, we have removed all instances of the word “young” from our writing. In addition, we added highlighted text to the conclusion to explicitly address the point that senior scientists/investigators can benefit from the considerations and points raised in this commentary as well.

Introduction

As basic and pre-clinical scientists, graduate students and trainees emphatically support the importance of both hearing and understanding the perspectives of individuals living with spinal cord injury (SCI); however, it is much more complex than most realize. Establishing avenues of communication between individuals with SCI, caretakers, and/or researchers is paramount to the success of our efforts in becoming well-rounded researchers that are knowledgeable about principles of human wellbeing. Here, we discuss the divides between researchers and individuals living with SCI that often create misunderstandings. By expanding on two topics within these divides, we seek to highlight the main message of this commentary from the student perspective: there is a need within the SCI research community for better data sharing practices and communication between pre-clinical researchers, clinicians, and individuals living with SCI. First, we will discuss diversity of data collection and sharing within the research realm.

To continue to produce relevant research, we must increase communication amongst researchers to boost accessibility with data sharing. Then, we will discuss relevant diversities in the SCI human population as we, young basic research scientists, understand them to exist 2 within the clinical setting. Such diversity is evident when diving into an individual’s background relating to culture, socioeconomic status, and education, which may all impact the care one seeks after sustaining an injury. By considering better data sharing practices as well as the variability and diversity within the human SCI condition, we and researchers at every level in their careers may better appreciate the complexity of the human condition after SCI and therefore ask questions with higher relevancy and value in our own research.

Data Sharing

For graduate students, a major focus of graduate training is publishing work in scientific journals as this is our formal path to most broadly communicating our research to the SCI community.

Unfortunately, a substantial amount of generated data may never be published or shared. This can be motivated by high publishing standards, grant proposal aspirations, and the desire to protect intellectual property. Furthermore, trainees in academia are rarely encouraged to engage in data sharing practices focused on non-scientific audiences beyond academia. This represents one example of a clear disconnect between scientific research and individuals living with SCI. As basic researchers, it can be challenging to even conceptually consider how experimental results can directly service or connect to the current population of individuals living with SCI. However, this is an important feature of SCI research that must be better incorporated into our academic research programs. It should be a core responsibility of basic and pre-clinical researchers to connect their work to the current population of individuals living with SCI as directly as possible. At the same time, scientific researchers have the responsibility to present high quality, verifiable, and reproducible findings in ways that prevent false narratives and minimize false hope for individuals living with SCI and their families. Clearly, data sharing is a complex duty for researchers.

Across SCI research labs, there is a lack of consistency in data collection practices. While this encourages independent critical thinking, it means our research community has much work to do in the pursuit of disseminating our data to the public in a consumable way. Great strides have been made toward promoting findable, accessible, interoperable, and reusable (FAIR) data practices within the basic and pre-clinical SCI research community in the form of a virtual open data repository (see Torres-Espín et al., 2021). The data from research labs that has been formatted, submitted, and ultimately uploaded into this FAIR database is then available for free download by anyone who has established a free account. This database is being advertised to clinicians, individuals living with SCI, and caretakers within the SCI community who may directly or indirectly benefit from information. We, as members of the SCI research community, must continue to take advantage of opportunities for outreach beyond the lab, such as conferences and retreats, where we can make meaningful connections with clinicians, individuals living with SCI, and other researchers. Our communities can benefit greatly from a better understanding of the needs of modern clinicians and individuals living with SCI, which are increasingly diverse and individualized. For example, the accessibility and needs of a spinal cord injured individual living with SCI in a rural community can vary drastically from those of someone in an urban setting. This diversity must be understood and considered in our pursuit of greater basic knowledge and more advanced therapies which positively impact individuals living with SCI.

Diversity and Accessibility Within the Current Population of Individuals Living with SCI

Within the current population of individuals living with SCI, there is a wide array of diversity within personal, family, and socioeconomic status (Khazaeipour et al., 2018). Because of this, 3 the population pool can have varying needs, goals, and desires that can have a substantial impact on care and quality of life (Scheel- Sailer et al., 2017). Furthermore, the efforts of researchers, physicians, and caretakers can obfuscate issues of data sharing, reproducibility, and consistency, resulting seemingly in a standstill in progress. As trainees representing the next generation of researchers in the field, we want to promote a better understanding of our work towards improving peoples’ lives, especially those who may be directly impacted by our research in the future. It is increasingly important to continually expand communication beyond the SCI research community. For these reasons, we are advocating for stronger direct connections between researchers and the members of the clinical SCI community. Specifically, we want to highlight the diversity of people with SCI and differences in accessibility to the most informed care amongst individuals because we believe that, in strengthening connections, individuals with SCI are the ones who should dictate what the priorities are for all members of the SCI community.

The impact that an individual’s education level, marital status, and occupation can have on their treatment plan is astronomical, which is further multiplied by the impact of these variables within one’s immediate family (Khazaeipour et al., 2018). Difficulties with choosing proper care, understanding treatment options, or even vocalizing their needs can make matters worse by adding to burdens beyond the injury itself (Scheel-Sailer et al., 2017). Such diversity amongst individuals living with SCI creates a difficult setting for physicians, researchers, and caretakers to appropriately support, as each individual living with SCI necessitates individualized care and attention.

Early after sustaining a SCI, one of the primary questions from individuals living with SCI is centered on “How long will it take for me to recover?”, quickly followed by “How will we afford this?”. As scientists, we are peripherally aware of the mental and financial burden that accompanies SCI, but rarely is there the opportunity to discuss and comprehend the full scope of injury. Furthermore, these burdens are often exacerbated by a lack of knowledge surrounding treatment options, the viability, and even feasibility of such risky ventures. These concerns have been voiced by some individuals living with SCI as having little to no knowledge about what clinical trials are available.

Embedded within all of this, ultimately, is a lack of adequate communication. Whether it be between caretakers, physicians, researchers, or individuals living with SCI, the inadequacy of communication is similarly exhibited across these issues. Relevant and updated communication is paramount as recovery, rehabilitation, and autonomy begins to take shape. Importantly, education and access to resources can change the perspectives of individuals living with SCI and their experience when taking control of their health care plans following SCI (Scheel-Sailer et al., 2017). This should not be one sided however, and there must be a balance between the learning experience for individuals living with SCI as well as those researching their ailments and treating or caring for them. As a pre-clinical researcher, these factors may seem daunting to consider. Where does one begin? We propose that in-person events focused on learning about and discussing the human condition are extremely valuable first steps. The points mentioned in this commentary can serve as talking points between clinicians, researchers, and individuals living with SCI to hopefully lead to a rich discussion of priorities, goals, and a broadened outlook on SCI research.

Conclusion

4

Even non-clinical research programs can sponsor events focused on the human experience after SCI. A good starting point for scientific researchers is to invite clinicians and individuals living with SCI to give or attend a presentation within your distinct research community. This can be as simple as a guest at a lab meeting, or a speaker at a department-wide seminar. In 2022, the Marion Murray Spinal Cord Research Center at Drexel University held its first annual NIH T32 sponsored retreat, entitled “Human Perspectives of Spinal Cord Injury.” It was attended by students, faculty, and individuals living with SCI. Jacob Chalfin, Chair of the SCI Advisory Committee for the state of Pennsylvania, gave the featured presentation entitled “A seat at the lab bench, leveraging the lived experience,” in which he discussed living with SCI in a way that trainees do not hear in classes, laboratories, or even many conferences. As basic research scientists, we were invited to broaden our perspectives throughout Jacob’s talk. Such exposure to different members of the SCI research community can then grow into more regular meetings, further internal discussions within the lab, and even long-term collaborations. At the very least, this exposure will provide a new perspective of the research we perform, which can oftentimes feel infinitesimal within the broader SCI community. It is important to recognize the ground-breaking program launched by Unite2Fight Paralysis called “Lab Rats” that is designed to bring individuals with SCIs into SCI research labs, to help address many of the deficiencies we noted in our training programs as basic science researchers.

As scientists, we recognize that increasing accessibility to research within the current population of individuals living with SCI and data transparency in general in the SCI research community represents goals worth striving towards. Currently, these endeavors are challenging within the existing frameworks of academia. However, reaching out to clinicians and individuals living with SCI is a relatively straightforward way to begin to form a more comprehensive network. This can be initiated by young scientists in research programs as well as more senior researchers and investigators, as pre-clinical researchers at any level in their career may benefit from connecting to the human perspective of SCI. These connections can only strengthen the field at large, and result in better understandings on all sides of the major goals and limitations associated with SCI research.
